# Comprehensive Classification System for Localized Alveolar Bone Deficiencies in Treatment Planning for Dental Implants: A Proposed Classification and Prevalence Study

**DOI:** 10.7759/cureus.67769

**Published:** 2024-08-25

**Authors:** Abhay Kolte, Rajashri Kolte, Pranjali Bawankar, Prachi R Rathi, Shreya Warkad, Pavan Bajaj, Prasad Dhadse

**Affiliations:** 1 Department of Periodontics and Implantology, Ranjeet Deshmukh Dental College and Research Centre, Nagpur, IND; 2 Department of Periodontics and Implantology, Sharad Pawar Dental College and Hospital, Datta Meghe Institute of Higher Education & Research, Wardha, IND

**Keywords:** dental implants, prevalence, classification, atrophy, alveolar ridge

## Abstract

Introduction

The current classification of alveolar bone defects remains ambiguous. This study aims to classify and evaluate the prevalence of bone deformities using a comprehensive classification system for localized alveolar bone deficiencies in dental implant treatment planning.

Methods

This cross-sectional prospective clinical trial included 698 participants (353 females and 345 males), patients with localized alveolar bone deficiencies. The clinical parameters evaluated were keratinized tissue width (KTW) and gingival thickness (GT) at the edentulous site. The width and height of alveolar bone deficiency at the site of implant placement were measured as horizontal deficiency (HD) and vertical deficiency (VD).

Results

Out of 698 patients, 566 (81.10%) had Subclass A horizontal deficiencies (HD), 99 (14.20%) had Subclass B HD, and 33 (4.70%) had Subclass C HD. Regarding vertical deficiencies (VD), 426 (61%) had Class I VD, 222 (31.80%) had Class II VD, and 50 (7.20%) had Class III VD. Younger individuals (20-30 years) predominantly exhibited Subclass A HD, whereas older participants (above 50 years) showed more severe deficiencies (Subclass B and C HD, and Class II and III VD). Gender analysis indicated no significant difference in HD prevalence but a significant difference in VD, with males more commonly presenting Class I VD and females exhibiting more Class II VD.

Conclusion

The study reveals significant associations between age and both HD and VD, indicating that older individuals tend to have more severe deficiencies. The study's findings underscore the importance of the proposed classification system in accurately identifying alveolar bone deficiencies and guiding appropriate treatment strategies, thereby improving clinical outcomes in dental implant therapy.

## Introduction

Alveolar ridge deformities in the oral cavity can result from periodontal breakdown, extractions, cysts, tumors, clefts, developmental anomalies, trauma, and congenitally missing teeth. Tooth extractions are the most common cause, often leading to reduced apico-coronal and buccolingual dimensions at the edentulous site. Research shows that post-extraction bone collapse is often three-dimensional (3D), but horizontal deficiency (HD) or width loss at the alveolar crest is more pronounced [1,2]. A deficit in alveolar width may indicate the loss of medullary and/or cortical bone in the buccal (labial) region, or both. Following tooth extraction, deficiencies in the cortical plate (buccal cortex) can provide serious challenges for implant restoration [3,4]. There seems to be an increased risk of future resorption for the buccal cortical plate with a thickness of less than 2 mm next to an implant [5].

Several authors have provided descriptions of the stages of alveolar ridge resorption following tooth removal [6,7]. Atwood (1971) identified six stages of alveolar ridge resorption after tooth extraction, from mild to severe [6]. Most bone loss occurs within the first year [7]. There is a 25% volume loss in the first year after extraction and a 40% reduction in three years. Prolonged edentulism leads to height deficiencies, while width deficiencies occur early due to the pattern of bone resorption.

Classification systems offer guidelines for treating specific clinical situations. They help define the clinical problem, guide treatment strategy, and set expectations for results. Understanding alveolar bone deficiency and suitable surgical procedures is essential before implementing these systems. Clearly explaining defect morphology improves patient awareness and acceptance of treatment, avoiding misunderstandings. However, existing classification systems do not adequately identify precise deformities or suggest appropriate treatments.

Seibert’s classification was first developed to address ridge deficiencies in preparation for obtaining a pontic [8]. Also, in order to improve the aesthetic look of fixed partial dentures, soft tissue augmentation techniques were the only treatment choices offered by this classification system. The classification by Wang and Al-Shammari (2002) attempts to categorize deficiencies in three dimensions, which is effective [9]. However, it does not consider adjacent anatomic structures or calibrate the defects [9]. This limitation makes it challenging to precisely quantify deficiencies, which is critical for deciding on treatment modalities. Later on, subsequent papers included the use of guided bone regeneration (GBR) to improve the osseous structures [10].

The proposed classification system uses stable anatomic landmarks such as the cemento-enamel junction (CEJ) of adjacent teeth and calibrates defects based on vertical, horizontal, or combined deficiencies. Accurate calibration helps clinicians choose the appropriate treatment modality. Additionally, assessing the prevalence of ridge defects according to this classification is essential. So, this study was planned to classify and evaluate the prevalence of bone deformities according to the proposed comprehensive classification system for localized alveolar bone deficiencies in treatment planning for dental implants.

## Materials and methods

This cross-sectional prospective clinical trial was performed from December 2022 to April 2024 in the Department of Periodontics and Implantology of our institute, Ranjeet Deshmukh Dental College and Research Centre (formerly VSPM Dental College and Research Centre), Nagpur. The protocol and design of the study were planned as per the guidelines of the Helsinki Declaration of 1975 and as revised in 2000 and presented to the Institutional Ethics Committee of VSPM (Vidya Shikshan Prasarak Mandal) Dental College and Research Centre, Nagpur which granted approval ethical clearance number IEC/VSPMDCRC/05/2022, dated January 15, 2022. The study was registered with the Clinical Trials Registry (CTRI/2022/09/045845). All participants were given written and verbal explanations of the study's goals, risks, and benefits prior to obtaining their written informed permission.

Patients visiting the Outpatient Department of Periodontics and Implantology with localized alveolar bone deficiencies with natural teeth on either side of the edentulous area seeking treatment with dental implant therapy were assessed clinically, on study models and digital panoramic radiographs.

Inclusion and exclusion criteria

The patients had to adhere to the inclusion criteria so as to be considered as study participants which were (a) patients between 20 to 60 years of age, (b) partially edentulous patients, (c) having natural teeth on either side of the edentulous area, and (d) patients having single or multiple edentulous areas within the mouth. The patients exhibiting the following clinical situations were excluded (a) they were completely edentulous, (b) patients who were treated surgically at the edentulous site, (c) patients who exhibited the presence of any tumur or cyst radiologically, and (d) patients who were on medications which would potentially influence the bone metabolism.

Clinical measurements

The clinical parameters evaluated included the keratinized tissue width (KTW) at the site of the edentulous area, and gingival thickness (GT) measured from the bone crest and labial side using a University of North Carolina (UNC)-15 periodontal probe (PCPUNC-15, Hu-Friedy Mfg. Co., LLC, Chicago, IL, USA). For measurement of KTW, the probe was positioned at the mucogingival junction and extended vertically to the crest of the ridge. The distance from the mucogingival junction to the crest of the ridge was measured and recorded as the KTW. The measurements were recorded to the nearest 0.1 mm.

For the measurement of GT, the edentulous ridge was examined, and specific points for measurement were marked, typically at the crest and labial side of the ridge. A calibrated periodontal probe was used to measure the GT. A topical anesthetic was applied at the measurement sites to minimize discomfort. The depth of insertion was noted, recording the thickness of the gingival tissue in millimeters. Each measurement was taken three times at each site to ensure accuracy. An average of the three measurements at each site was calculated to obtain a final GT value and KTW for each measurement point. By adhering to this standardized method, consistency and reliability in measuring KTW and GT in edentulous areas were maintained across all participants.

After the study models were prepared the patients were taken up for panoramic radiograph to assess the adjacent teeth and alveolar bone condition. The following radiographic parameters were evaluated in these patients: (1) Width of alveolar bone deficiency at the site of implant placement (horizontal deficiency, HD): The width of the alveolar ridge was measured clinically 3 mm apical from the crest of the gingival margin at the site of implant placement with the use of soft tissue caliper and then categorized based on the classification; (2) Height of alveolar bone deficiency at the site of implant placement (vertical deficiency, VD): The height of VD of alveolar bone was measured radiographically on a digital panoramic radiograph at the site of implant placement from the CEJ of the mesial and distal tooth (a mean of both these measurements was taken as the final measurement of VD); (3) Based on the above measurements and according to the comprehensive classification system for localized alveolar bone deficiencies in treatment planning for dental implants which is described in Table 1 and Table 2, the deficiencies were classified. Any other bone deficiency in vertical and/or horizontal directions that is uneven in nature and does not fall under the described classes was categorized as Class IV.

**Table 1:** Localized vertical alveolar bone deficiency. CEJ: cemento-enamel junction.

Classification	Description	Suggested treatment
Class I	Vertical bone deficiency in an apico-coronal direction not exceeding 3 mm from the CEJ of the adjacent mesial and distal teeth.	Such types of deficiencies may not need any treatment or at the most to enhance aesthetics a soft tissue augmentation procedure may be carried out.
Class II	Vertical bone deficiency in an apico-coronal direction from 3.1mm to 6mm from the CEJ of the adjacent mesial and distal teeth	Such types of deficiencies can be treated with particulate bone grafts along with guided bone regeneration procedures.
Class III	Vertical bone deficiency in an apico-coronal direction more than 6mm from the CEJ of the adjacent mesial and distal teeth.	Such types of deficiencies are severe in nature and can be corrected by vertical bone augmentation through block autografts or allografts. Particulate bone graft materials can additionally be used for secondary grafting on the lateral aspects of the block graft.

**Table 2:** Localized horizontal alveolar bone deficiency. CEJ: cemento-enamel junction.

Classification	Description	Suggested treatment
Subclass A	Horizontal ridge dimensions in a facio-lingual or bucco-lingual direction above 6mm at the CEJ of the adjacent mesial and distal teeth.	Such types of deficiencies may not require any treatment as the horizontal dimensions can suitably house an implant of smaller dimensions.
Subclass B	Horizontal ridge dimensions in a facio-lingual or bucco-lingual direction from 3mm to 6mm at the CEJ of the adjacent mesial and distal teeth.	Such types of deficiencies can be treated with a bone expansion procedure with sequential osteotomes and may at times require the use of particulate bone graft materials and barrier membranes in case of exposure of a few threads of dental implant.
Subclass C	Horizontal ridge dimensions in a facio-lingual or bucco-lingual direction less than 3mm at the CEJ of the adjacent mesial and distal teeth.	Such types of deficiencies are severe in nature and can be corrected by horizontal bone augmentation through block autograft or allograft. Particulate bone graft materials can additionally be used for secondary grafting on the lateral aspects of the block graft.

All the above parameters were assessed by two separate examiners SW and RK who were calibrated prior to the beginning of the study. Based on the class of the alveolar ridge deficiency, another two periodontists, AK and PB, were asked to suggest the treatment protocol for each of the deficiencies. Inter-examiner calibration and agreement were crucial aspects of this study to ensure the reliability and consistency of the measurements. Before the commencement of data collection, all examiners underwent a thorough calibration process, which involved training sessions and practice measurements on a subset of participants. This was done to align their techniques and criteria for assessing the key parameters, including VD, HD, KTW, and GT from both the crest and labial side. The calibration process was followed by an agreement assessment using intra-class correlation coefficients (ICCs) to evaluate the consistency between examiners. High ICC values (>0.9) indicated excellent inter-examiner reliability, ensuring that the measurements were accurate and reproducible across the entire study population.

Statistical analysis

The descriptive statistics including the gender distribution, HD, and VD were expressed as frequency and percentage distribution. The descriptive statistics for the clinical parameters like KTW, GT from the crest, and GT from the labial side were expressed as the mean and standard deviation. The Chi-square test was used to check the significance of the association between age groups and HD and VD. All the analyses were performed using the software IBM SPSS Statistics for Windows, Version 29.0.2 (IBM Corp., Armonk, NY), and statistical significance was tested at 5% level.

## Results

Out of 698 participants (353 females and 345 males), the age distribution was as follows: 204 individuals (29.2%) were aged 20-30 years, 243 individuals (34.8%) were aged 31-40 years, 165 individuals (23.6%) were aged 41-50 years and 86 individuals (12.3%) were above 50 years. The mean KTW for the entire study population was 2.35 ± 0.87 mm, GT from the crest was 3.10 ± 0.53 mm, and GT from the labial side was 2.00 ± 0.35 mm. The mean KTW was found to be 2.20 mm in females and 2.50 mm in males.

Out of the total number of observations in the dataset 566 individuals (81.10%) had Subclass A HD, 99 (14.20%) had Subclass B HD, and 33 (4.70%) had Subclass C HD as depicted in Table 3. Regarding vertical defects as shown in Table 3, 426 participants (61%) had Class I VD, 222 (31.80%) had Class II VD, and 50 (7.20%) had Class III VD.

**Table 3:** Horizontal and vertical deficiency.

Horizontal deficiency	Frequency	Percent
Subclass A	566	81.10
Subclass B	99	14.20
Subclass C	33	4.70
Total	698	100.00
Vertical deficiency	Frequency	Percent
Class I	426	61.00
Class II	222	31.80
Class III	50	7.20
Total	698	100.00

**Figure 1:**
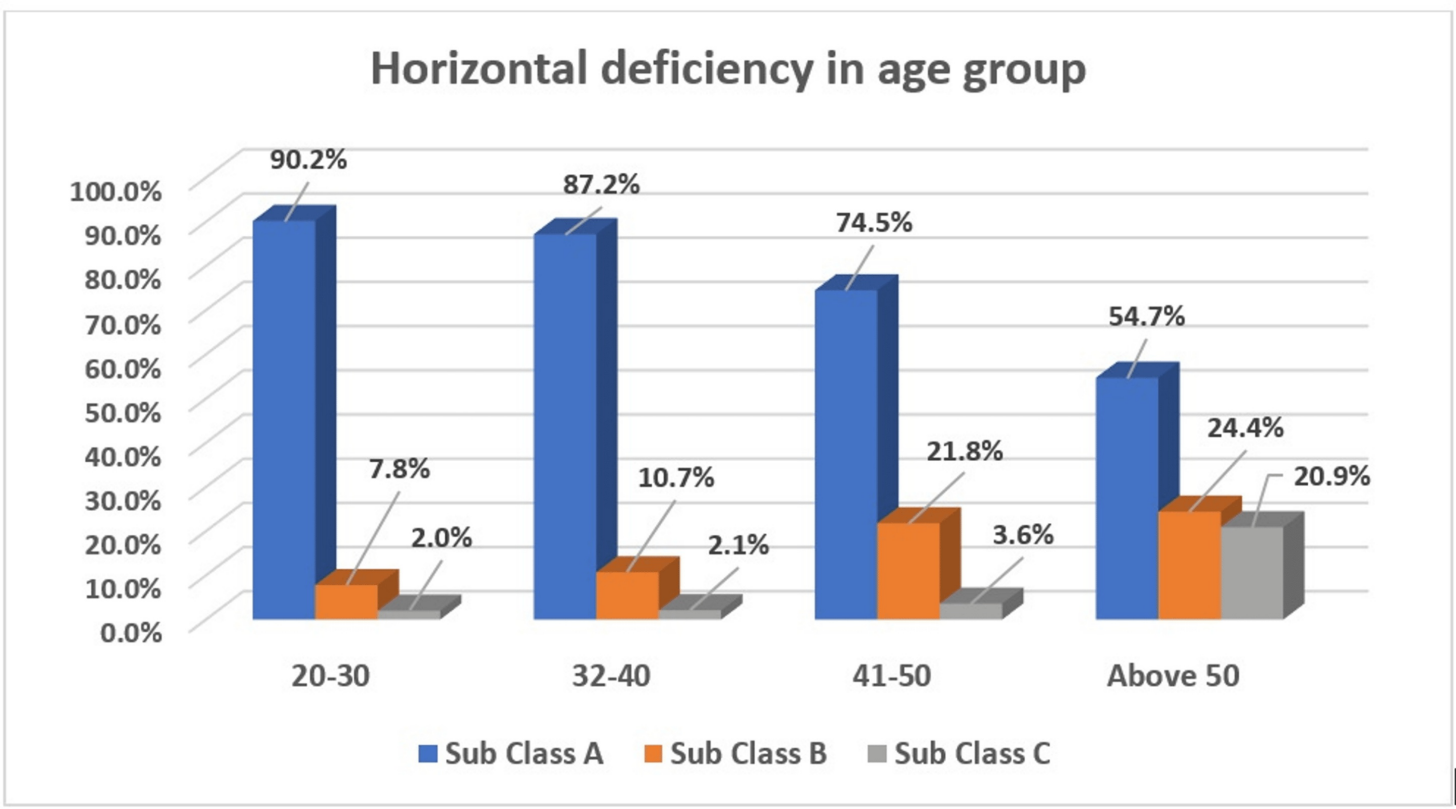
Percentage distribution of horizontal deficiencies in different age groups.

Figure 1 shows the age-wise distribution of the horizontal bone deficiencies revealing that in the 20-30 age group, 90.2% had Subclass A, 7.8% had Subclass B, and 2.0% had Subclass C. For the 31-40 age group, the distribution was 87.2% for Subclass A, 10.7% for Subclass B, and 2.1% for Subclass C. In the 41-50 age group, 74.5% had Subclass A, 21.8% had Subclass B, and 3.6% had Subclass C. For those above 50, 54.7% had Subclass A, 24.4% had Subclass B, and 20.9% had Subclass C. The Chi-square test confirmed a significant difference between age and HD.

**Figure 2:**
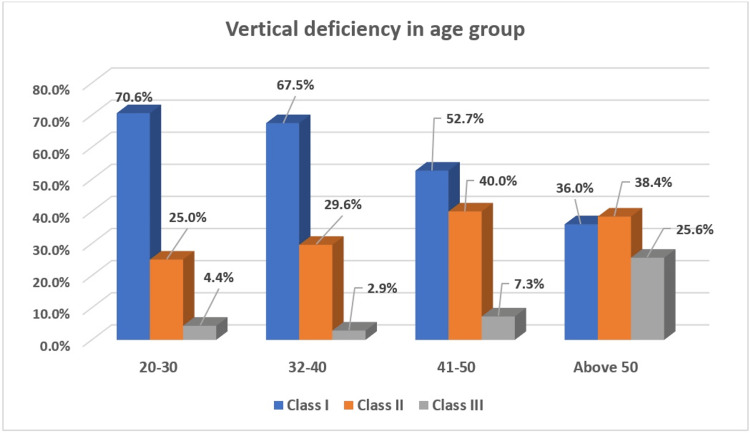
Percentage distribution of vertical deficiencies in different age groups.

Figure 2 illustrates the distribution of VD across different age groups and includes Chi-square test results to determine the significance of the association between age groups and VD. In the 20-30 age group, 70.6% had Class I deficiency, 25.0% had Class II, and 4.4% had Class III. For the 31-40 age group, 67.5% had Class I, 29.6% had Class II, and 2.9% had Class III. Among the 41-50 age group, 52.7% had Class I, 40.0% had Class II, and 7.3% had Class III. In the above 50 age group, 36.0% had Class I, 38.4% had Class II, and 25.6% had Class III. Overall, 61.0% of the sample had Class I deficiency, 31.8% had Class II, and 7.2% had Class III. The Chi-square test yielded a p-value of 0.000, indicating a statistically significant difference between age groups and VD.

Table 4 exhibits the distribution of HD across different genders. Among the participants, 289 females (51.1%) and 277 males (48.9%) exhibited Subclass A HD. For Subclass B deficiencies, 47 females (47.5%) and 52 males (52.5%) were affected. Subclass C deficiencies were observed in 17 females (51.5%) and 16 males (48.5%). The Chi-square test produced a p-value of 0.80, indicating insignificant differences between gender and HD.

**Table 4:** Horizontal deficiency in male/female. p-value: probability value, 0.80 > 0.05 (not significant).

Deficiency	Gender		
Horizontal deficiency	Female	Male	Total
	289	277	566
Subclass A	51.1%	48.9%	100.0%
	47	52	99
Subclass B	47.5%	52.5%	100.0%
	17	16	33
Subclass C	51.5%	48.5%	100.0%
	353	345	698
Total	50.6%	49.4%	100.0%

Table 5 presents the distribution of VD by gender, along with Chi-square test results. Among the total study population 197 females (46.2%) and 229 males (53.8%) had Class I type of VD while 133 females (59.9%) and 89 males (40.1%) had Class II and 23 females (46.0%) and 27 males (54.0%) exhibited Class III type of VD. The Chi-square test yielded a p-value of 0.03, indicating a statistically significant difference between gender and VD.

**Table 5:** Vertical deficiency in male/female. p-value: probability value, 0.03 < 0.05 (significant).

Deficiency	Gender		
Vertical deficiency	Female	Male	Total
	197	229	426
Class I	46.2%	53.8%	100.0%
	133	89	222
Class II	59.9%	40.1%	100.0%
	23	27	50
Class III	46.0%	54.0%	100.0%
	353	345	698
Total	50.6%	49.4%	100.0%

## Discussion

The present study assessed the prevalence of bone deficiencies using a comprehensive classification system for localized alveolar bone deficiencies in dental implant treatment planning. This research is crucial for effective ridge augmentation procedures. Understanding the prevalence of various bone defects in both partially and completely edentulous patients helps clinicians assess the clinical landscape, especially in the context of planning and executing successful dental treatments. Given the high complication rates of appositional bone graft techniques, like graft integration and stability, poor vascularization, risk of infection, extended healing time, soft tissue coverage and tension-free closure, graft resorption, etc. More complex defects, such as those with significant vertical or horizontal bone loss, are inherently more challenging to treat and have higher complication rates. Proper categorization of these defects helps in planning and predicting outcomes. Hence, categorizing these defects by complexity is vital. This will help in procedure selection. Different degrees of vertical and horizontal bone loss require different treatment approaches. For example, minor deficiencies might be addressed with simpler procedures like bone grafts or guided bone regeneration, while more significant deficiencies may need complex surgical interventions such as ridge augmentation or distraction osteogenesis. The study aimed to determine the prevalence and complexity of localized alveolar bone defects to facilitate effective treatment planning.

The demographic analysis of our study population of 698 participants, comprised of 353 females and 345 males, represents a balanced gender distribution. The included participants were the highest percentage in the 31-40 year age group (34.8%), followed by the 20-30 year age group (29.2%). Participants aged 41-50 years and those above 50 years comprised 23.6% and 12.3% of the sample, respectively. This diverse age distribution allows for a complete examination of the relationship between age and bone deficiencies.

Our findings reveal that Class I VD was the most common, followed by Class II and Class III deficiencies. Similarly, Subclass A HD was more prevalent than Subclasses B and C. Notably, 90.2% of younger individuals aged 20-30 years exhibited Subclass A HD. In contrast, Subclasses B and C were more frequently observed in individuals aged over 50 years. The majority of the participants exhibited Subclass A HD (81.10%), while 14.20% had Subclass B HD, and 4.70% had Subclass C HD. The prevalence of HD across different age groups showed a clear trend confirming a significant difference between age and HD and suggesting that older age groups are more likely to exhibit severe HD. This trend highlights the progressive nature of HD with advancing age. Similar results were reported in previous studies [11,12], which suggest the prevalence and treatment of different classes of vertical bone deficiencies, highlighting that Class I deficiencies are frequently encountered and treated in clinical practice compared to more severe Class II and III deficiencies. Gender analysis revealed no statistically significant difference in HD prevalence (p-value = 0.80). Subclass A HD was almost equally prevalent among females (51.1%) and males (48.9%). Similarly, Subclass B and C deficiencies were balanced between genders, indicating that HD is influenced more by age than by gender.

VD was categorized into Class I, II, and III which demonstrated an overall distribution comprising Class I VD which was the most prevalent (61%), followed by Class II (31.80%) and Class III (7.20%). The Chi-square test yielded a p-value of 0.000, indicating a significant difference between age groups and VD. This suggests that as age increases, the severity of VD also increases, reflecting the cumulative impact of aging on vertical bone health.

Gender analysis exhibited a significant difference in VD prevalence with Class I VD being more common in males (53.8%) than females (46.2%). Conversely, Class II VD was more prevalent in females (59.9%) than males (40.1%), while Class III VD was slightly more frequently observed in males (54.0%) than females (46.0%). These findings suggest potential gender-specific factors influencing the severity of VD. The higher prevalence of VD and HD in younger individuals compared to the older may be attributed to several factors, including differences in bone metabolism, a more robust inflammatory response, and accelerated bone resorption. These observations align with the findings of Das et al. (2020), reinforcing the notion that age-related variations in bone resorption patterns are significant [13].

A study by Lang et al. (2013) suggested that younger individuals tend to have a higher bone turnover rate, which, while being beneficial for repair and remodeling, can also lead to increased bone resorption when coupled with inflammatory conditions [14]. This heightened bone turnover may result in more significant bone deficiencies in younger populations.

The results of the present study also reveal that Subclass C and Class III type of bone deficiencies and combined Class II Subclass III or Subclass III or vice versa defects were prevalent among individuals above 50 years of age. The probable reason for this is older individuals are more likely to experience greater VD and HD due to several age-related factors. A primary reason is the decline in bone density and regenerative capacity with age, mainly due to decreased osteoblast activity, impairing new bone formation, and sustained or increased osteoclast activity, leading to bone resorption. Ghezzi and Ship (2000) highlighted that age-related changes in bone metabolism significantly contribute to periodontal bone loss [15]. Aging reduces the body's responsiveness to inflammatory stimuli, fostering chronic inflammation and subsequent bone degradation. Additionally, older adults often contend with comorbidities like osteoporosis and diabetes, which exacerbate bone loss. Long-term exposure to risk factors such as smoking, poor oral hygiene, and periodontal disease also play a cumulative role in substantial bone loss over time. Papapanou and Susin (2017) reported the impact of periodontitis on bone health, particularly in older adults, and addressed the increased vertical and horizontal bone deficiencies associated with chronic periodontal disease in this population, underscoring the impact of chronic periodontal disease on bone health in the elderly [16].

Gender comparison showed no significant association with HD. However, significant gender differences were found in VD, particularly among middle-aged females, who exhibited a higher prevalence of Class II VD. This may be attributed to hormonal changes during menopause, leading to decreased estrogen levels critical for bone density maintenance. Estrogen deficiency accelerates bone resorption and decreases bone formation, leading to a higher risk of osteoporosis and subsequent bone deficiencies. According to a study by Khosla (2010), postmenopausal women experience rapid bone loss due to the reduction in estrogen, which affects both cortical and trabecular bone [17]. Additionally, middle-aged women may face an increased prevalence of periodontal diseases, which can contribute to bone loss. The study by Reinhardt et al. (1999) also corroborated these findings [18].

In the present study, it was found that males demonstrated more Subclass II type of HD as compared to females. This can be attributed to several factors, including lifestyle habits, differences in bone metabolism, and systemic health conditions. Men are more likely to engage in behaviors that negatively impact bone health, such as smoking and excessive alcohol consumption. These habits are known risk factors for periodontal disease and bone loss. A study by Dietrich et al. (2007) found that smoking is strongly associated with alveolar bone loss, and males are more likely to be smokers compared to females, contributing to a higher prevalence of HD [19]. Males and females have different bone remodeling dynamics. Although males generally have a higher peak bone mass, they also experience a more gradual but continuous bone loss with age. This continuous loss can result in more pronounced HD over time. Seeman (1995) discussed the mechanism of males experiencing trabecular thinning which can lead to HD [20]. Our graphs suggest exactly this predilection suggesting the accuracy and reliability of the new proposed classification.

Gender-specific analysis showed that males had a slightly higher mean KTW (2.50 mm) compared to females (2.20 mm). These differences, while slight, could have clinical implications in terms of periodontal health and treatment approaches. The fundamental knowledge and basis for defining suitable hard and soft tissue dimensions for implant placement are considered to be clinical expertise, along with the physical and mechanical prerequisites for the implant placement procedure. Most of the authors recommend a minimum width of 5 mm and a height of 7 to 10 mm of bone [21,22]. Various ridge augmentation treatments, including block grafts, particle grafts, GBR (guided bone regeneration), ridge expansion techniques, and distraction osteogenesis, have been described for the enhancement of both height and width in cases of insufficient ridges [23-27].

In 1985, Allen et al. offered a modification to Siebert's categorization that took the ridge's magnitude into account [26]. The purpose of this classification was to help with the prognosis and treatment planning for patients with alveolar ridge defects. Another classification system proposed by Lekholm and Zarb included bone resorption, from minimal to severe, and classified it into five categories [10]. Similar to this, cross-sections of the alveolar process resorption level are presented in Cawood and Howell's ridge categorization [28]. The biggest shortcoming of previous classification systems [6,8,28,29] is the fact, that the two-dimensional representations of those classes fail to convey the three-dimensional nature of atrophic ridges.

Regretfully, aesthetics is not taken into account in this classification system. The alveolar ridge defect that occurs due to anterior tooth loss, is concerning because it is challenging to correct owing to aesthetic considerations [30]. Implant rehabilitation serves as more than merely a means of regaining lost phonological and masticatory abilities but also aesthetically acceptable and structurally sound [31].

The proposed classification systems of ridge volume represent valuable guidelines for evaluating alveolar ridge defects. This classification system takes into account stable anatomic landmarks such as the CEJ of the teeth adjacent to the edentulous areas and calibrates the defects according to the vertical, horizontal, or combined deficiencies followed by probable treatment modality for each of these defects. It is proposed that accurate calibration of the deficiency enables the clinician to decide precisely about the suitable treatment modality to be adopted in a specific situation.

## Conclusions

The study found significant age-related associations with both horizontal deficiency (HD) and vertical deficiency (VD), indicating greater severity in older individuals. Gender differences were not significant for HD but were observed for VD, suggesting biological or behavioral factors at play. These findings underscore the utility of the proposed classification in diagnosing and managing HD and VD for oral rehabilitation. Class I and Subclass A ridge defects were most prevalent. The classification system, based on anatomical and radiological parameters, prescribes specific treatment strategies, facilitating collaboration among specialists in treatment decisions.
